# Classification of Four-Class Motor Imagery Employing Single-Channel Electroencephalography

**DOI:** 10.1371/journal.pone.0098019

**Published:** 2014-06-20

**Authors:** Sheng Ge, Ruimin Wang, Dongchuan Yu

**Affiliations:** 1 Key Laboratory of Child Development and Learning Science of Ministry of Education, Research Center for Learning Science, Southeast University, Nanjing, Jiangsu, China; 2 School of Electronic Engineering and Optoelectronic Technology, Nanjing University of Science and Technology, Nanjing, Jiangsu, China; University of Rome Tor Vergata, Italy

## Abstract

With advances in brain-computer interface (BCI) research, a portable few- or single-channel BCI system has become necessary. Most recent BCI studies have demonstrated that the common spatial pattern (CSP) algorithm is a powerful tool in extracting features for multiple-class motor imagery. However, since the CSP algorithm requires multi-channel information, it is not suitable for a few- or single-channel system. In this study, we applied a short-time Fourier transform to decompose a single-channel electroencephalography signal into the time-frequency domain and construct multi-channel information. Using the reconstructed data, the CSP was combined with a support vector machine to obtain high classification accuracies from channels of both the sensorimotor and forehead areas. These results suggest that motor imagery can be detected with a single channel not only from the traditional sensorimotor area but also from the forehead area.

## Introduction

The brain-computer interface (BCI) is a new communication scheme that depends on neither the brain's normal output nerve pathways nor the muscles. Using a BCI system, one can directly translate brain activities into sequences of control commands for an output device such as a computer application [1], [2]. Motor imagery is a mental process by which an individual rehearses or simulates a given action in his/her mind but without actually producing movement; it is assumed to involve similar cortical areas that are activated during actual motor preparation and execution [3]. Motor imagery has been widely used as a major approach in BCI studies [4], [5].

In most BCI research, whole-head multi-channel data are used to produce high accuracy. However, the large number of electrodes required implies a longer time spent in channel preparation. In addition, the BCI system may be expensive as many amplifiers are needed. As BCI research has advanced, portable systems with fewer channels have become essential in applying BCIs to everyday life and home applications. The preparation of the electrodes involves putting gel or paste on the scalp and fitting an electroencephalography (EEG) cap on the head. Additionally, the skin needs to be prepared to deal with the hair under the electrodes. By comparison, in long-term daily-life BCI usage, it is much easier to fit EEG electrodes on the forehead area because there is no hair in this area. Additionally, it is inconvenient and uncomfortable to place multiple electrodes on the scalp. A realistic solution is to place a few electrodes or a single electrode over the motor cortex or, since it is easier and more comfortable to place electrodes on the forehead to get the motor imagery signal from the forehead if possible. Thus, in this study, we hypothesize that if high classification accuracy can be obtained in motor imagery tasks using only a few EEG channels or a single EEG channel from forehead electrodes, then the use and application of a motor-imagery BCI system will be much easier and more convenient.

Our hypothesis must address how to extract adequate and appropriate features of motor imagery from a system comprising few or a single channel. The common spatial pattern (CSP) method is commonly used for effective feature extraction [6]–[9]. The main idea of CSP method is to use a linear transform to project multi-channel EEG data into a low-dimensional spatial subspace with a projection matrix, of which each row consists of weights for channels. However, CSP can only be effectively used if there are many electrodes available [10]. Therefore, it is not appropriate to use CSP for a few- or single-channel system.

Some previous research has focused on single-channel electrocorticography BCI [11]. Müller-Putz et al. reported success in detecting foot motor imagery (one-class) employing single-channel EEG [12]. Pfurtschellers group [13], [14] used one Laplacian channel (signals from the surrounding electrodes were used) to detect motor imagery. Some multi-channel BCI research has also attempted single-channel analysis, but the signals from the remaining channels were used during the analysis [15], [16].

In this paper, we propose a method of using only single-channel EEG data to classify four-class motor imagery. We first decompose the single-channel EEG signal into the time-frequency domain. In the time-frequency domain, we treat the frequency band as a variable, and we thus have multi-channel time-varying inputs. With this transformation, the original single-channel input can be transformed into a multi-channel input. Therefore, CSP can be used in feature extraction. To the best of our knowledge, this research is the first to address four-class motor-imagery BCI with a single-channel EEG.

## Methods

### Data acquisition

**Figure 1:**
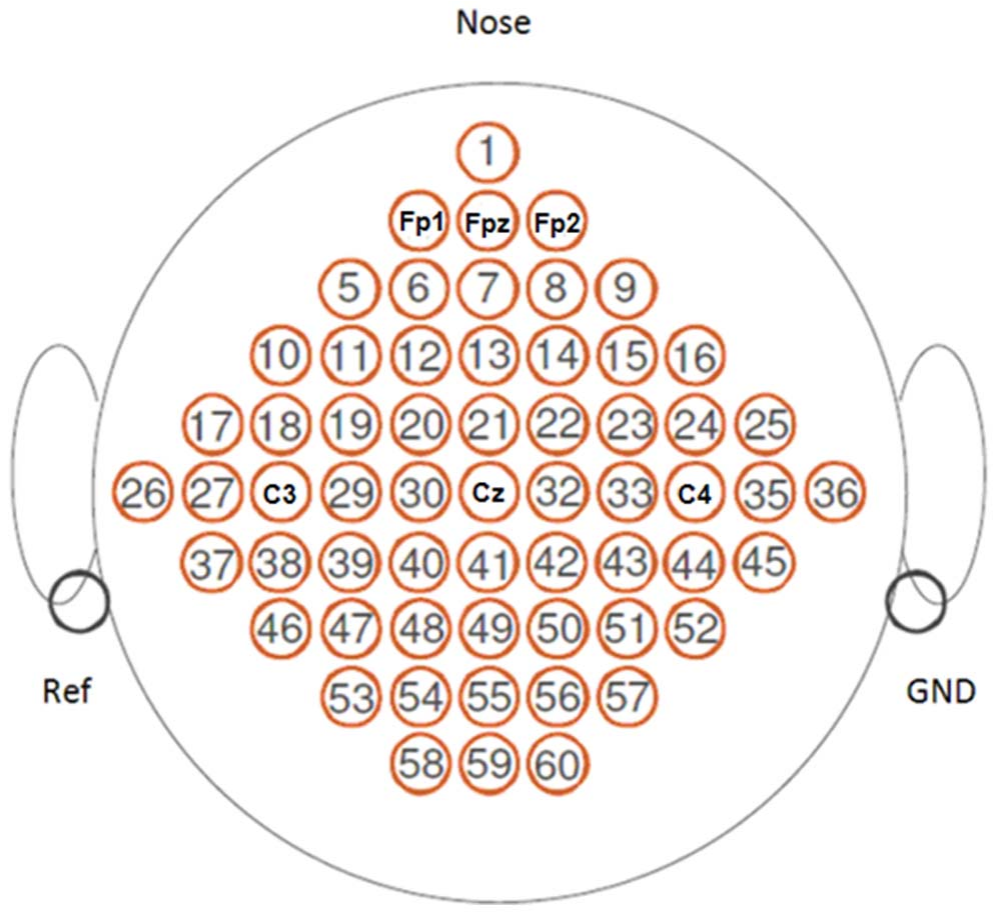
Position of EEG electrodes.

In our retrospective study, we used the dataset IIIa from the 2005 BCI competition provided by the University of Technology, Graz, Austria [17]. All participant records and information used in this study were anonymous and were not identified in the dataset. The Ethics Committee of Southeast University approved our study protocol and methods before we conducted this research. This dataset comprises 60-channel EEG data for a four-class (left hand, right hand, foot, and tongue) classification task. The datasets were recorded for three participants, K3, K6 and L1, using a Neuroscan EEG amplifier. The left mastoid served as a reference and the right mastoid as the ground. The EEG was sampled at 250 Hz and filtered between 1 and 50 Hz. A notch filter allowed suppression of line noise. Sixty EEG channels were recorded according to the scheme in Figure 1.

**Figure 2:**
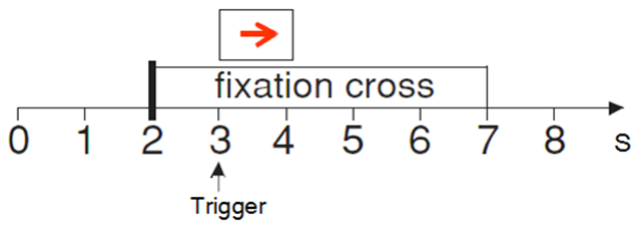
Timing of the paradigm.

The participants were seated relaxed in a chair with armrests, and were instructed to perform imaginary movements prompted by a visual cue. Each trial started with an empty black screen; at time point 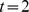 s a short beep tone was presented and a cross + appeared on the screen to catch the participants attention. Then, at 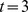 s, an arrow appearing for 1.25 s pointed either to the left, to the right, upwards or downwards. Each position indicated by this arrow instructed the participant to imagine a left hand, right hand, tongue or foot movement, respectively. The respective imaginary movement was to last until the cross disappeared at 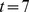 s (see Figure 2). The data set recorded from participant K3 consisted of 9 runs, whereas the data sets from K6 and L1 consisted of 6 runs each. Each of the four cues was displayed 10 times within each run in a randomized order, and each trial lasted for 7 s. Trials with labels, which indicated that the trials had visually identified artifacts, were excluded from the input data for analysis.

### EEG electrode selection

Previous knowledge tells us that the C3, Cz and C4 electrodes, which are over the sensorimotor area, record important characteristics of motor imagery [18], [19]. In this study, we selected EEG data from C3, Cz and C4. Moreover, Fp1, Fpz and Fp2, which are over the forehead, were also used in this study.

### Time-frequency analysis

The purpose of this study was to distinguish four-class motor imagery only using single-channel EEG data. Therefore, it was important to extract more information from single-channel data. In this study, we employed time-frequency analysis to obtain both temporal and frequency characteristics. By performing time-frequency analysis, a single time-varying signal can be converted into multiple time-varying signals at different frequencies. Such a channel-increasing method allows past multi-channel BCI approaches, such as the use of CSP, to be applied to the single-channel case.

Short-time Fourier transform (STFT) analysis, wavelet transform (WT) analysis and Hilbert-Huang transform (HHT) analysis are the most used time-frequency analysis methods. The time resolution of the WT is hundreds of milliseconds, with a central frequency below 20 Hz [20], while past motor imagery research has reported that the mu (8–13 Hz) and beta (16–25 Hz) rhythms served as effective classification features to distinguish motor imagery [21], [22]. Further, empirical mode decomposition in HHT analysis often encounters such problems as mode mixing and ending effect, and is very sensitive to noise [23]. Compared with these two methods, STFT analysis has acceptable time and frequency resolution below 20 Hz. The most important point is that the calculation cost of the STFT is far lower than those of the WT and HHT. Thus, the STFT is a reliable method for BCI analysis. In this study, we used the STFT (spectrogram function of Matlab's Signal Processing Toolbox) for time-frequency analysis of single-channel EEG data, while a 50% overlapped Hamming window of size 128 samples was used, and the number of FFT 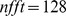 samples (each 100 original samples were zero-padded to 128 points). Since the mu (8–13 Hz) and beta (16–25 Hz) frequency bands play a key role in classification of motor imagery [21], [22], the 8–30 Hz frequency band was investigated.

### Feature extraction

In most BCI research, the CSP is widely used to separate two different classes. The idea behind using such a binary CSP is to find an optimal decomposition to transform two classes of data into a common space, in which the two classes of transformed data have the same principal components, and their corresponding eigenvalues add up to a unit matrix. The idea behind the CSP is to find a spatial filter that can be applied such that the projected signal has high power for one class and low power for the other. Here, the power in a trial is calculated using the variance in the time domain. The binary CSP can discriminate only between two different classes (e.g., left versus right). For k-class paradigms, an extension has been proposed [24], [25]: the basic idea is to decompose the k-class problem into a set of k binary problems (right versus rest, left versus rest, etc.). Each problem consists of discriminating one class against the remaining classes (one versus the rest, OVR) [26].

Here, we will derive an OVR algorithm for the four-class case. We denote the STFT matrices 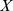 of a single-channel EEG signal for four different directions as 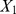, 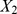, 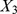 and 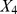 with dimensions of 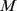 by 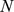, where 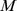 and 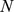 are the numbers of frequency and time bands, respectively. The spatial covariance of STFT matrices for these conditions can therefore be estimated by
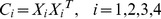
(1)where 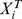 denotes the transpose of 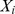. As for the binary CSP, we can build the composite covariance matrix as
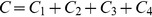
(2)


The composite covariance matrix can be factored by eigen-decomposition as
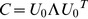
(3)where 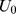 is the 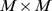 unitary matrix of principal components, and 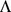 is the 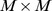 diagonal matrix of eigenvalues.

The whitening transformation matrix is then formed as
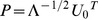
(4)


To see how to extract common spatial patterns specific to condition 1, we let
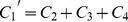
(5)



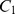
 and 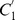 are then individually transformed as
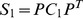
(6)

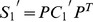
(7)


It can be demonstrated that 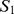 and 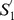 share common principal components [24]. If the eigen-decomposition of can be written as
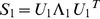
(8)where 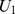 is the eigenvector matrix of 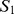, which corresponds with eigenvalue matrix 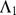. Then 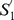 can be factored as
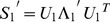
(9)and the sum of the corresponding eigenvalue matrices 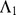 and 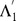 will be a unit matrix:
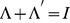
(10)


Combining equations (4) and (6)–(10), we have
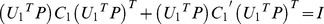
(11)



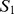
 and 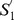 share common eigenvectors and the sum of corresponding eigenvalues for these two conditions will always be one.

From equation (11), the variance accounted for by the eigenvectors corresponding to the 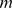 largest eigenvalues will be maximal for 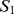, and minimal for 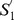. Therefore, the transformation of the STFT matrix 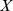 onto eigenvector space will maximize the variance difference between 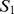 and 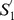. The projection matrix 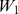 is
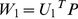
(12)


A 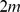-by-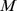 spatial filter 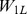 was built with the first and last 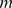 rows of 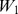. Then, the STFT matrix 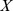 is filtered with this spatial filter:
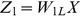
(13)


The filtering of the STFT matrix 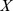 leads to a new time-frequency matrix 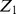. The pattern is designed such that the 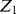 that results from the 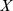 filtered with 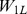 has maximum variance for 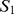 and minimum variance for 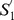. In this way, we can extract the common spatial patterns specific to 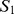; i.e., condition 1.

In the same way as above, we can build spatial filters 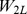, 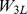 and 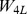 to get the filtered time-frequency matrices 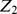, 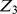 and 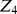 for the remaining conditions 2, 3 and 4, respectively.

### Classification

Feature vectors for four different conditions are obtained:
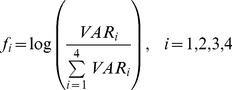
(14)where 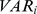 is the variance of 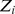 among time points (1-by-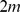). A composite feature vector (1-by-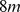) is defined as:
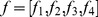
(15)


As a state-of-the-art classification methodology, the support vector machine (SVM) [27] has sound theoretical foundations and has served as a powerful tool for solving classification problems [28]. With respect to the recognition of a small sample of nonlinear and high-dimensional data, SVM has better adaptability, stronger classification ability and higher computational efficiency. In this study, we used the LIBSVM package [29] to implement SVM classification, and traditional C-support vector classification (C-SVC) [30] was used as the support vector classifier.

The basic idea of SVM is to look for the optimal decision hyperplane that best separates the data points into different classes with a maximum margin, while allowing errors during separation; i.e., map the input 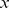 onto a high-dimensional feature space (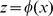) and construct an optimal hyperplane defined by 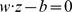 to separate examples into different classes, where 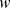 is the normal vector and 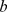 is the bias of the separation hyperplane. This is done by solving the primal problem:
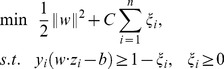
(16)where 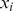 is the 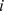-th input sample, 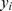 is the class label value corresponding to 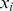, 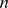 is the number of input samples, 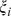 is the slack variable that allows an example to be in the margin (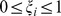, also called a margin error) or to be misclassified (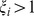), and 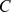 is a penalty factor.

The equation (16) can be solved by its dual problem using Lagrange optimization; i.e., we solve the quadratic programming (QP) problem
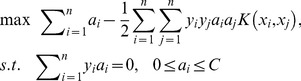
(17)where 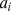 is the Lagrange multiplier from the QP problem, and 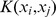 is the kernel function.

Because of the nonlinear properties of EEG signals, in this study, the radial basis kernel function (RBF) is selected as the SVM kernel function:
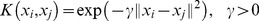
(18)where 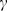 is the kernel parameter. The kernel parameter 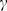 and penalty factor 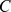 are the main parameters that affect the performance of the SVM. 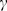 decides the distribution of the transformed data in space, and the penalty factor 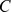 controls the degree of punishment for right or wrong classification, thus balancing classification violation and the margin. Therefore, 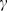 and 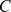 play an important role in improving the correct rate and classification efficiency of the SVM. In this study, the grid search method [31] was used to optimize 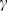 and 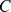. To prevent the overfitting problem, we used a 10×10-fold cross-validation procedure. In this procedure, the training set is divided into 10 subsets of equal size. Sequentially, one subset is tested using the classifier trained on the remaining nine subsets. The optimal 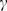 and 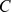 are obtained when the cross-validation accuracy is a maximum. The final classification accuracy is the mean result of the 10-fold cross-validation procedure.

## Results

**Figure 3:**
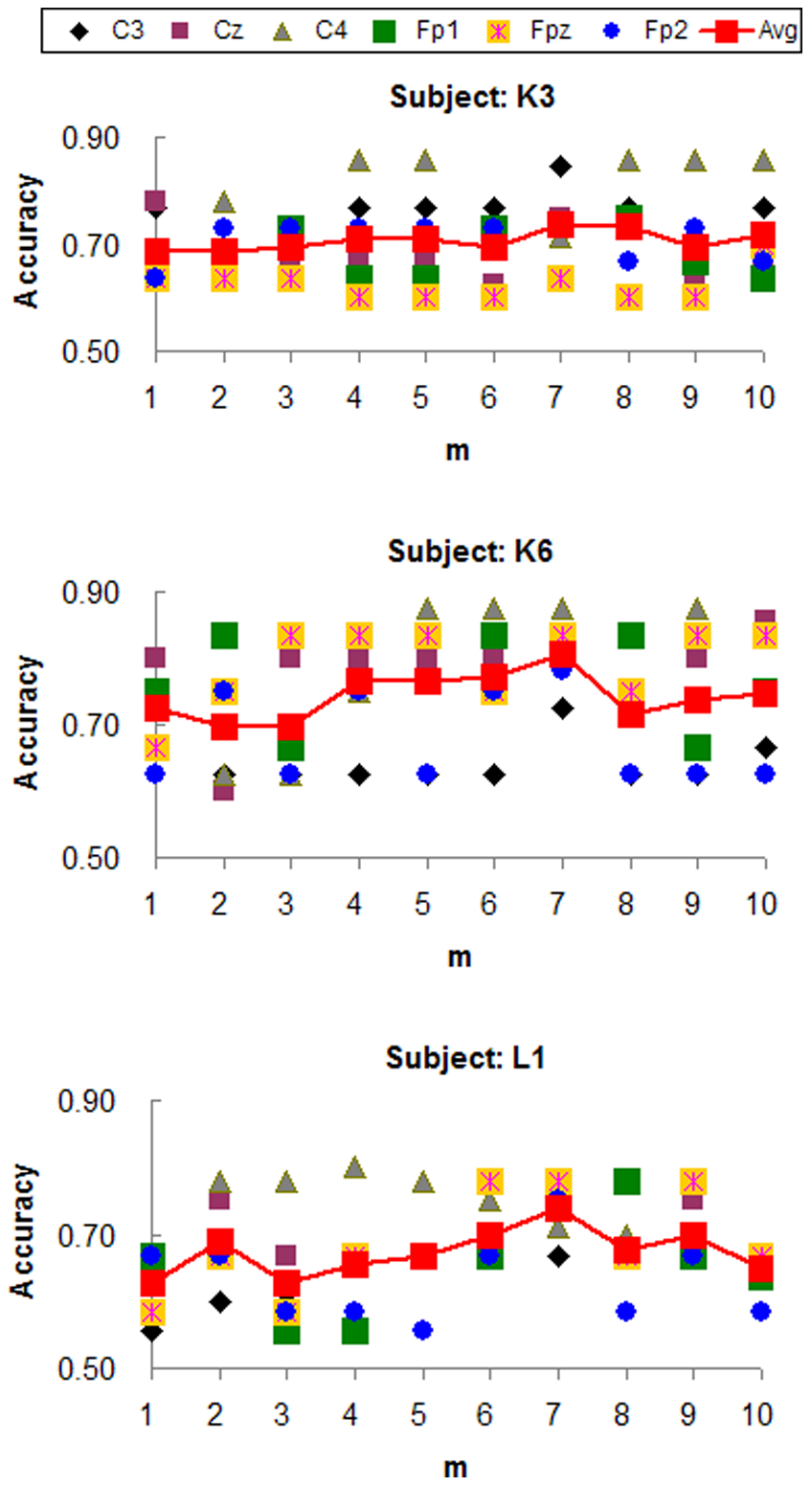
Accuracy values at different m values for participants K3, K6 and L1. The mean accuracy value peaked at m = 7 for all three participants.

The main free parameter affecting the classification accuracy is 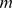, which is the number of projections to CSP used to build the feature vector. The classification accuracies for participants K3, K6 and L1 with different 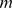 values were compared in the range from 1 to 10 (see Figure 3). According to the curve of averaged accuracy, it was clear that the classification accuracy peaked when 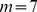 for all three participants.

Table 1 presents accuracy values for different time ranges and electrodes for participants K3, K6 and L1. The time ranges are set as four different ranges: 3∼4, 4∼5, 5∼6 and 6∼7 s. Table 1 also gives accuracy values for different EEG electrodes (i.e., Fp1, Fpz, Fp2, C3, Cz and C4) and for all the three participants. Two-way analysis of variance (ANOVA) was employed to investigate the effects of the time range and electrode selection. There were no significant difference for either the time range (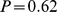) or the electrode selection (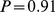).

**Table 1:** Accuracy values for different time ranges and electrodes for participants K3, K6 and L1.

			Time Range	
CH	Ppt	3∼4s	4∼5s	5∼6s	6∼7s	Avg
Fp1	K3	0.52	0.74	0.58	0.58	0.63
	K6	0.63	0.83	0.63	0.63	
	L1	0.55	0.58	0.56	0.78	
Fpz	K3	0.53	0.60	0.64	0.58	0.62
	K6	0.55	0.83	0.53	0.63	
	L1	0.78	0.58	0.67	0.54	
Fp2	K3	0.56	0.64	0.73	0.67	0.65
	K6	0.63	0.78	0.51	0.63	
	L1	0.55	0.75	0.67	0.67	
C3	K3	0.64	0.85	0.69	0.64	0.64
	K6	0.56	0.73	0.50	0.63	
	L1	0.50	0.58	0.67	0.67	
Cz	K3	0.75	0.60	0.56	0.56	0.64
	K6	0.80	0.60	0.60	0.57	
	L1	0.56	0.67	0.63	0.75	
C4	K3	0.71	0.64	0.67	0.70	0.65
	K6	0.88	0.63	0.63	0.63	
	L1	0.56	0.50	0.63	0.71	
Avg		0.62	0.67	0.61	0.64	

## Discussion

Past work [7], [15], [32]–[36] used few- or single-channel EEG data to classify four-class motor imagery using the 2005 BCI competition dataset, which was used in our study. We list the classification accuracy results obtained in these studies in Table 2.

**Table 2:** Best accuracy for different feature-extraction and classification methods.

Feature Extraction	Classifier	Channel	Accuracy(%)		
			K3	K6	L1
AAR	MDA	Best single channel of 60 ch [38]	56.9	46.5	48.5
AAR	MDA	Three best single channels of 60 ch [38]	66.6	38.5	49.5
CAR+CSP	NN	C3 and C4 [44]	41.6	41.7	49.5
Barlow method	SVM	C3 and C4 [12]	53.3	42.5	55.8
Barlow method	SVM	C3, Cz and C4 [12]	63.3	45.0	60.0
WPD	ME	C3, Cz and C4 [14]	90.8	66.0	76.9
WPD+CSP	SVM+NN	C3, Cz and C4 [27]	83.1	84.4	85.6
PLV	SVM+Quicksort	C3, Cz and C4 [22]	86.0	82.0	77.0
Sparse PCA+Sparse CSP	SVM	60 ch [39]	85.1	81.6	80.1
STFT+CSP	SVM	Fp2 [our Method]	73.4	78.3	75.2
		C4 [our Method]	71.3	88.1	71.2

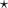
Since there are four classes of imagery movements, the chance level is 25.

AAR: adaptive autoregressive; MDA: minimum distance analysis; CAR: common average reference; CSP: common spatial pattern; NN: neural network; SVM: support vector machine; WPD: wavelet packet decomposition; ME: mixture of experts; PLV: phase-locking value; PCA: principal component analysis; STFT: short-time Fourier transform.

Most past research used more than two electrodes to extract features. Only Schlogl et al. [15] used the best single channel of 60 EEG channels for classification. However, since they used all 60 channels of data and then picked the best single channel, their method differs from that of using only single-channel information to detect motor imagery.The best classification result in past research was obtained by Li et al. [35], who used three combined channels and got 83.1, 84.4 and 85.6% for participants K3, K6 and L1, respectively.

Unlike past research that used at least two combined channels or selected the best channel from multiple channels, our method used only single-channel data to get 73.4, 78.3 and 75.2% from the Fp2 channel, and 71.3, 88.1 and 71.2% from the C4 channel for each participant respectively. This result is relatively better than the results of most of the previous studies.

Although the averaged accuracy for the 4∼5 s time range was considerably higher than that for other time ranges, the ANOVA result showed that there were no significant differences in other time ranges. Wang et al. [32] found that the best accuracy for different participants was obtained for different time ranges. Our result supports their conclusion.

The ANOVA result for electrode selection verified that there were no significant differences in accuracies obtained with the electrodes used in this study. The accuracies obtained using C3, Cz and C4, which are over the sensorimotor area, were equivalent to those obtained using Fp1, Fpz and Fp2, which are far from the motor cortex. As a result of volume conduction [37], the local EEG activity field also produces a far-field potential [38] and the active potential will not only be recorded directly above the generator but will also appear as a function of current spreading over the skull and scalp [39]. Fried et al. [40] reported that the P14 component, which is generated by the parietal lobe, can be similarly recorded by both parietal and frontal lobe electrodes. Nunez [41] reported high coherence of EEG channels over large distances. More directly, Li et al. [42] found high correlation in the event-related potential, frequency domain and event-related spectral perturbation between forehead-area EEGs and sensorimotor-area EEGs during a motor imagery task. These past studies and our result confirm that forehead EEG electrodes can be used to detect motor imagery equally as well as using traditional electrodes over the sensorimotor area.

The CSP algorithm has been shown to be one of the most popular and efficient algorithms for BCI detection [6]–[9]. A disadvantage of the CSP method is the large number of electrodes needed [10]. The accuracy will be poor if the number of electrodes is insufficient [43]. In this study, employing the STFT, we transformed the time domain signal of a single channel into multiple frequency-domain signals. If we treat such multiple frequency-domain signals as a form of multi-channel information, the CSP can be applied to single-channel EEG. Using the STFT, the time-domain signal is converted to a time-frequency domain signal. Thus, the one-dimensional feature in the time domain is expanded to two-dimensional features in the time-frequency domain. Past studies have shown that the frequency feature plays an important role in BCI detection [14], [35], [43], [44]. Our method expands time features to time and frequency features, allowing more feature vectors to be used in feature detection. In this study, we used such a method to examine the classification accuracies of different single electrodes. The results demonstrate that expansion of a single time-domain signal to multiple frequency-domain signals is an efficient approach to obtain high classification accuracy of motor imagery with a single-channel EEG.

## Open Questions

Compared with the traditional motor imagery research that is based on sensorimotor area EEGs, detecting motor imagery based on forehead area EEGs is a novel approach. From the perspective of convenience and comfort, forehead-type BCI systems may be highly possible and practical for usage in everyday life in the future. However, forehead area EEGs also inevitably involve electrooculography (EOG) and electromyography (EMG) signals. BCI research must ensure that it is only EEG signals, but not EOG or EMG signals, that play a key role in classification. Although, we had already tried to reduce EOG and EMG effects in our research by excluding visually identified artifacts, more research and discussion about this problem based on a large number of data is needed in the future.

In this research, we selected sites C3, C4 and Cz near the sensorimotor area, which are considered to have a relationship to motor imagery and are widely used in BCI studies. Moreover, considering usage in everyday life, we also selected Fp1, Fp2, Fpz at the forehead area, which are easy to locate and set. Although higher classification accuracies were obtained from those electrodes in this study, it is hard to conclude that those electrodes are the optimum channel(s) for all other participants. Our research has just shown that these electrodes would be good candidates for single-channel BCI system.

Another limitation of this study is that the dataset from the 2005 BCI competition that is used in this research only contains 3 participants. Further verification with more datasets is needed to demonstrate the robustness of our proposed method.

## Conclusions

In this study, we applied STFT to decompose single-channel EEG signal into the time-frequency domain to construct multi-channel information. Based on these reconstructed data, we used CSP combined with a SVM to obtain equivalent high classification accuracies from both the sensorimotor and forehead areas, which suggests that motor imagery can be detected with a single channel not only from the traditional sensorimotor area but also from the forehead area.
